# Mechanical axis image quality of the EOS imaging system compared to digital radiographs

**DOI:** 10.1007/s00256-026-05264-6

**Published:** 2026-06-13

**Authors:** Ahmed Alghamdi, Natalie Lee, Farag Shuweihdi, Amaka C. Offiah

**Affiliations:** 1 Division of Clinical Medicine, University of Sheffield, Western Bank, Sheffield, UK; 2 Radiologic Sciences Department, College of Applied Medical Sciences, Kingdom of Saudi Arabia, King Khalid University, Abha, Kingdom of Saudi Arabia; 3 Radiology Department, Sheffield Children’s NHS Foundation Trust, Western Bank, Sheffield, UK; 4 Leeds Institute of Health Sciences, University of Leeds, Leeds, UK

**Keywords:** EOS imaging system, Digital radiographs, Mechanical axis imaging

## Abstract

**Objective:**

To compare mechanical axis image quality between the EOS imaging system and digital radiograph (DR).

**Materials and methods:**

This retrospective study included 100 patients who underwent both EOS and DR mechanical axis imaging. It was conducted according to the Quality and Standards Department at the University of Sheffield. Three observers independently scored the DR and EOS images according to the modified European guidelines on quality criteria for diagnostic radiographic images in paediatrics: resolution, coverage of the area of interest, positioning, contrast, and motion. The range of scores was (Yes) for the fulfilled criterion and (No) for the not fulfilled criterion. For the motion criterion, the scores were (No) if the image quality criterion was not fulfilled (motion) and (Yes) if the image quality criterion was fulfilled (no motion). Inter-rater agreement was measured using the Gwet’s agreement coefficient (AC1) with a 95% confidence interval. The accuracy of the EOS was assessed using sensitivity, specificity, and the area under curve (AUC). Variables between the EOS and DR were compared using McNemar’s test. Both Gwet’s AC1 and corresponding 95%CI were computed using irrCAC library in R4.41 software.

**Results:**

The mean (range) age of patients when they underwent DR and EOS imaging was 11.2 (4–18) and 11.8 (5–17) years, respectively. EOS demonstrated almost perfect inter-rater reliability in the resolution criterion (AC1 = 0.99, 95% CI = 0.98,1.00, *p* < 0.001), while DR showed similar reliability in the motion artefacts (AC1 = 0.96, 95% CI = 0.92.00, *p* < 0.001). Intra-rater reliability was high for most criteria. EOS images demonstrated high sensitivity for all criteria and variable specificity, which equalled or exceeded that of DR images. Significant differences were found in some criteria, such as the coverage of the area of interest (chi-squared = 37.39, *p* < 0.001) and hip level (chi-squared statistic = 31.44, *p* < 0.001).

**Conclusion:**

The high AC1, sensitivity, and AUCs highlight the diagnostic image quality of the EOS imaging system for mechanical axis images, making it a valuable modality in clinical settings. Therefore, the EOS imaging system can be used instead of DR in this context, offering advantages such as lower irradiation, shorter scanning time, and the capability for 3D reconstruction.

## Introduction

Lower extremity malalignment and unequal leg lengths are significant orthopedic disorders requiring attention [1, 2]. An adequate and symmetrical lower extremity alignment contributes to establishing good posture and appropriate gait, without which individuals may suffer pain and develop early osteoarthritis [3, 4].

**Fig. 1:**
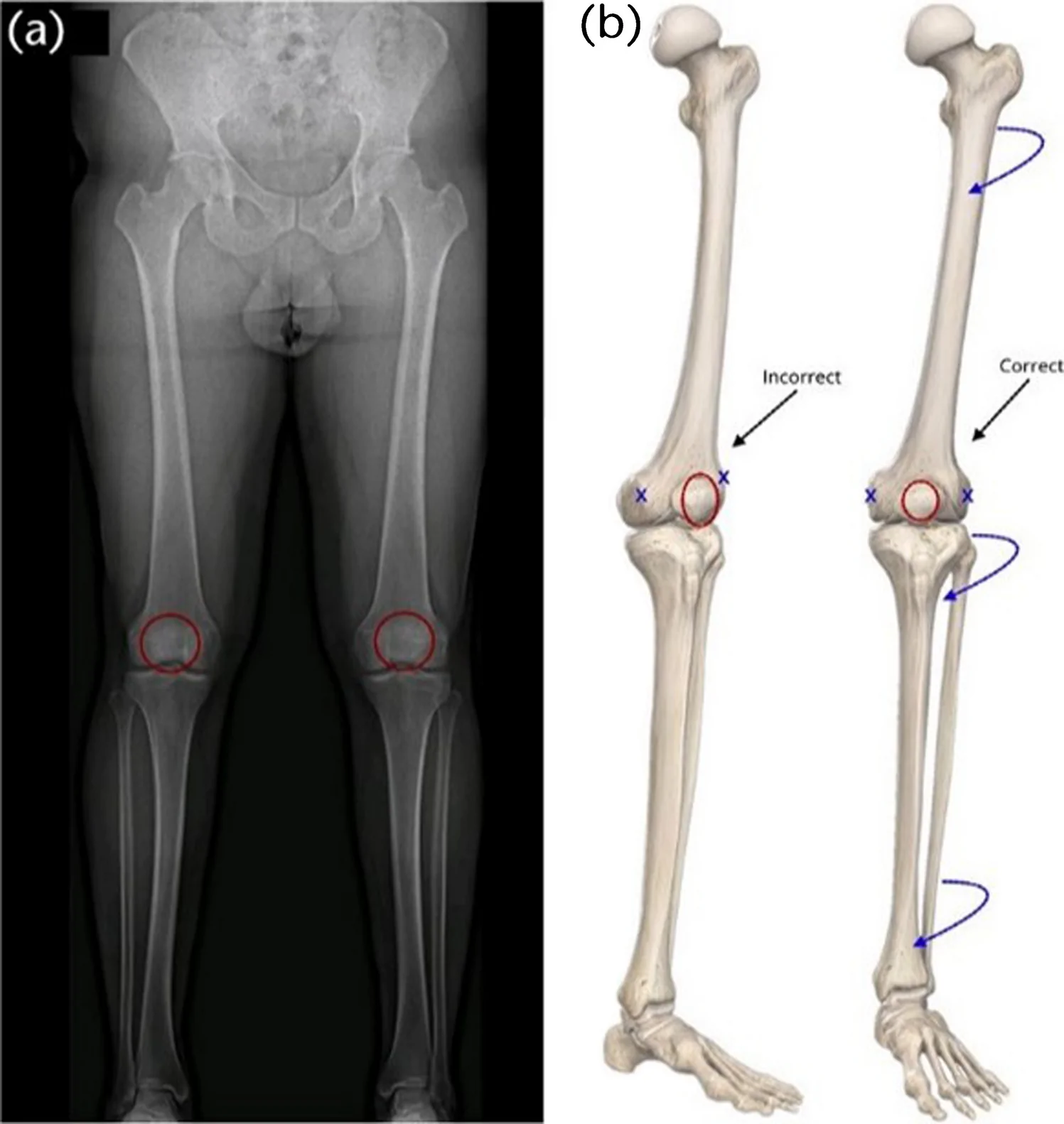
Assessment of the mechanical axis on full-length anteroposterior (AP) radiographs and 3D anatomical reference. **a** Radiographic image showing the mechanical axis of the lower limb, defined as a line drawn from the center of the femoral head to the center of the ankle joint. In normal alignment, this line passes through or within ± 6 mm of the center of the knee joint (red circles). **b** 3D anatomical illustration demonstrating correct (right) and incorrect (left) mechanical axis alignment relative to the knee center, highlighting the clinical importance of proper rotational and positional alignment in lower extremity assessment

The mechanical axis assesses lower extremity alignment and is obtained by a line drawn from the centre of the femoral head to the centre of the ankle joint, normally passing through the center of the knee joint (± 6 mm) Fig. 1 [5]. The mechanical axis image can be used to identify unequal limb lengths [6, 7]. Lower extremity measurements are important factors to consider when evaluating individuals with malalignment of the mechanical axis [8, 9].

Imaging modalities are essential in assessing lower extremity malalignment and unequal limb lengths. Identifying the positions of the ankle and hip is essential for accurately evaluating the mechanical and anatomical axes of the lower extremities [5]. Digital radiograph (DR) can evaluate spine and lower extremity malalignment and unequal limb lengths. A full-length standing anteroposterior (AP) radiograph is the reference method for evaluating the lower extremities [7, 10], with the advantages of accessibility, low cost, and short imaging times [4, 11, 12]. Despite such advantages, it has disadvantages that may limit its use, such as a higher radiation dose than other imaging modalities (e.g., the scout view of computed tomography [CT] scans), image magnification, and artefacts [4]. Another imaging modality that can be used for such measurements is CT. However, imaging patients in the supine position might affect the assessment accuracy of limb alignment [9].

The EOS imaging system, a new biplanar radiography device, was recently introduced, comprising two co-linked pairs of X-ray tubes and detectors that move vertically to simultaneously capture two-dimensional (2D) AP and lateral images that can be reconstructed into three-dimensional (3D) images [13–15]. The most crucial benefit of this modality is its lower radiation doses than an X-ray and CT [16–18]. Since patients are placed in a weight-bearing position during the EOS scan (an advantage for functional measurements), images are likely to contain motion artefacts, especially in patients who cannot remain stable [19, 20].

The complexity and ambiguity of the term “image quality” may pose challenges in quantitative evaluation. Despite technical and physical parameters, observer experience and perception are crucial parameters in image quality evaluation [21]. Various types of visual grading, including both subjective and objective methods, can be used to evaluate the reproduction of essential anatomical structures based on the quality criteria outlined in the European Commission Guidelines [22]. This study uses one of these methods, the fulfilment of image criteria, in which observers assess whether a radiograph meets specific criteria. An image criteria score is then calculated based on the proportion of criteria fulfilled [23]. Although the assumption of equal importance for all criteria is a limitation, the approach was chosen for its simplicity and consistency, with the understanding that more complex weighted systems could be explored in future research.

After an EOS imaging system was installed at Sheffield Children’s NHS Foundation Trust in 2018, a consultant orthopedic surgeon reached the subjective opinion that its mechanical axis image quality was inferior to that of DR. The referring orthopaedic surgeon’s assessment of “inferior” image quality was based on their clinical experience and the ability to accurately interpret images for diagnostic and measurement purposes. The term “inferior” referred to specific aspects of the image quality that the surgeon felt could potentially impact their ability to make accurate measurements. These concerns included reduced sharpness of key anatomical landmarks and/or insufficient contrast in certain regions critical for evaluating mechanical alignment. These issues were not necessarily severe enough to prevent measurement but may have introduced uncertainties in specific cases. It has been stated that it is unacceptable to judge the quality of a new modality as inferior based on overall image appearance; instead, it should be assessed based on several factors, such as diagnostic accuracy and technical capacity (e.g., contrast and resolution) [24]. Therefore, this study compared the mechanical axis image quality of the EOS imaging system and DR.

## Materials and methods

This retrospective study was conducted according to the Quality and Standards Department at the University of Sheffield. Informed consent was not required since the images were retrospectively reviewed. It examined the full-length standing AP radiographs of 100 patients who had undergone both EOS and DR mechanical axis imaging. Images (analyzed over six months) were randomly selected from the picture archiving and communication system of the Radiology Department at the Sheffield Children's NHS Foundation Trust.

The DR images were obtained using two systems: the Agfa DR 600 system with a DX-D 40 C wireless detector (Hamburg, Germany) and the Philips BuckyDiagnost X-ray system (Hamburg, Germany). The patients were asked to stand upright, with their hip, femurs, patellae, and ankles aligned in the AP position. The EOS images were obtained using an EOS imaging system (EOS Imaging, Paris, France). The patients were in the weight-bearing position, holding the bar at their chest level, with their feet placed on the landmark of the EOS floor.

Three observers (a pediatric musculoskeletal radiologist with 22 years of experience, a pediatric radiographer with 10 years of experience, and a radiographer with 5 years of experience) independently scored the DR and EOS images on two separate occasions (eight weeks apart) according to the modified European guidelines on quality criteria for diagnostic radiographic images in paediatrics [22]. They assessed the fulfilment of the following image criteria: resolution (the visibility of trabecular pattern/bony outline); coverage of the area of interest (if the image involved the area from the pelvis to ankles); positioning (if the hips were at the same level based on the top of the femoral head and iliac crest and if patellae faced forward); contrast (noticeable difference between muscle and fat); and motion artefacts (obscuring in the radiographs). The mechanical axis radiographs were subjectively assessed according to the abovementioned criteria. The scores were (Yes) if the image quality criterion was fulfilled and (NO) if the image quality criterion was not. For the motion criterion, the scores were (No) if the image quality criterion was not fulfilled (motion) and (Yes) if the image quality criterion was fulfilled (no motion).

### Statistical analysis

Inter-rater agreement was assessed using Gwet’s agreement coefficient (AC1) with a 95% confidence interval (CI). Gwet’s AC1 is less sensitive to prevalence bias. When the prevalence of specific categories is very high or very low, Cohen’s kappa can produce artificially low agreement scores, even when actual agreement is high. Gwet’s AC1 adjusts for this by accounting for the probability of chance agreement in a manner less influenced by category prevalence. AC1 is a statistical measure used to assess inter-rater reliability, addressing limitations of Cohen’s kappa, particularly its sensitivity to prevalence and marginal distributions. While both AC1 and kappa adjust for chance agreement, AC1 estimates chance agreement differently, assuming raters classify items independently, which provides more stable and reliable results when category prevalence is unbalanced. It is calculated using the observed agreement and an adjusted expected agreement and is commonly applied in studies involving categorical ratings by multiple observers. A 95% confidence interval was reported to indicate the precision of the estimate. The commonly used thresholds for Cohen’s kappa can be applied to Gwet’s AC1 as a guideline: 0.81–1.00: almost perfect agreement; 0.61–0.80: substantial agreement; 0.41–0.60: moderate agreement; 0.21–0.40: fair agreement; 0.01–0.20: slight agreement; and 0.00 or below: poor or no agreement. We measured inter-rater agreement using the entire sample of 100 cases to ensure a comprehensive agreement assessment between raters across all data. To assess intra-rater agreement, we randomly selected 33 cases from the same sample. This random selection allows us to balance practicality while providing sufficient statistical power to detect consistency within raters. The diagnostic summaries of the EOS were assessed using sensitivity, specificity, and the area under the receiver operating characteristic curve (AUC). McNemar’s test was used to compare the significant differences between EOS and DR. We assumed an arbitrary level of 5% statistical significance (2-tails). The statistical software used was the IBM Statistical Package for Social Sciences (SPSS), Version 28.0 (Armonk, NY, IBM Corp.). Both Gwet’s AC1 and corresponding 95%CI were computed using irrCAC library in R4.41 software. The diagnostic performance of a test can be summarized using a standard contingency table (Table 1).

**Table 1:** Diagnostic performance of a test

Test result	Disease present	Disease absent
+ ve	True positive (a)	False positive (b)
−ve	False negative (c)	True negative (d)

From this table, several important indices are derived:

- Total sample size = (a + b + c + d) = i.
- Disease prevalence = (a + c)/n.

Test sensitivity (Se) refers to a test’s ability to correctly identify individuals who have the disease. It is calculated as: Se = *a*/(*a* + i).

Test specificity (Sp) refers to a test’s ability to correctly identify individuals who do not have the disease. It is calculated as: Sp = *d*/(*b* + *d*).

Positive predictive value (PPV) is the probability that an individual who tests positive actually has the disease. PPV is positively related to disease prevalence: as prevalence increases, PPV also increases, because the likelihood that a positive test represents a true positive rises.

Negative predictive value (NPV) is the probability that an individual who tests negative truly does not have the disease. In contrast to PPV, NPV decreases as disease prevalence increases, since a greater proportion of negative results may represent false negatives at higher prevalence levels.

The general formulas for predictive values at any given prevalence are as follows:

- PPV for *any* prevalence = Se × Sp/Se × prevalence + (1−Sp) × (1−prevalence).
- NPV for *any* prevalence = Sp × (1−prevalence)/(1−Se) × prevalence + Sp × (1−prevalence)_._

It is important to note that predictive values are heavily influenced by disease prevalence in the population being tested. Therefore, PPV and NPV calculated in one population may not be generalizable to other populations with different prevalence rates. This limitation restricts the direct application of predictive values outside the original study context. Area under the receiver operating characteristic curve (AUC) is the probability of being in a diseased group compared to a non-diseased one. The probability (AUC) ranges from 0.5 (no discrimination) to 1.0 (perfect discrimination) between the two groups. A straight line is drawn at 45% to give the area distinction. A 95% confidence interval can be estimated; this gives precision. AUC plots sensitivity (*y*-axis) versus 1−specificity (*x*-axis). As the curve sweeps upwards and toward the left, the AUC becomes higher. 1−specificty is preferred to specificity because of aesthetics: It looks prettier. A plot of sensitivity vs. specificity would be a mirror image with no change to the AUC or its interpretation. Covariate adjustment can be accommodated via logistic regression.

## Results

### Inter-rater agreement with EOS and digital radiograph

Table 2 Presents the inter-rater agreement for the EOS and DR. Inter-rater agreement varied in strength across the image criteria scores for the DR. It was highest for motion artefacts (AC1 = 0.96, 95% CI = 0.92, 1.00, *p* < 0.001), indicating almost perfect concordance. It was also almost perfect for resolution (AC1 = 0.91, 95% CI = 0.85, 0.96, *p* < 0.001) and contrast (AC1 = 0.90, 95% CI = 0.83, 0.97, *p *< 0.001). It was substantial for the coverage of the area of interest (AC1 = 0.85, 95% CI = 0.77, 0.92, *p* < 0.001) and moderate for the hip level (AC1 = 0.72, 95% CI = 0.61, 0.83, *p* < 0.001). It was the lowest but still substantial for the patellae facing forward (AC1 = 0.64, 95% CI = 0.52, 0.77, *p* < 0.001). Overall, these results indicate that the DR provides reliable inter-rater agreement for most assessments, with particularly strong agreement in motion artefacts and resolution.

**Table 2:** Inter-rater agreement of the European criteria as applied to EOS and DR. Agreement coefficients with 95% confidence intervals (CI) and *p*-values are reported for each criterion. High inter-rater agreement was observed for both modalities across most criteria, with near-perfect agreement for motion and resolution in DR (coefficients ≥ 0.91) and similarly strong agreement in EOS. The lowest agreement was observed for the “patellae facing forward” criterion in both DR (0.64) and EOS (0.76), though all agreement coefficients were statistically significant (*p* < 0.001)

Criterion	Agreement coefficient (95%CI) (DR)	*p*-value	Agreement coefficient (95%CI) (EOS)	*p*-value
Motion	0.96 (0.92, 1)	< 0.001	0.84 (0.77, 0.91)	< 0.001
Resolution	0.91 (0.85, 0.96)	< 0.001	0.99 (0.98, 1.00)	< 0.001
Contrast	0.90 (0.83, 0.97)	< 0.001	0.95 (0.90, 0.99)	< 0.001
Coverage of the area of interest	0.85 (0.77, 0.92)	< 0.001	0.85 (0.77, 0.92)	< 0.001
Hip level	0.72 (0.61, 0.83)	< 0.001	0.68 (0.57, 0.80)	< 0.001
Patellae facing forward	0.64 (0.52, 0.77)	< 0.001	0.76 (0.664,0.86)	< 0.001

Inter-rater agreement varied in strength across various image criteria scores for the EOS. It was very high for resolution (AC1 = 0.99, 95% CI = 0.98,1.00, *p* < 0.001), indicating nearly perfect agreement. It was almost perfect for contrast (AC1 = 0.95, 95% CI = 0.90, 0.99, *p* < 0.001). It was almost perfect for the coverage of the area of interest (AC1 = 0.85, 95% CI = 0.77, 0.92, *p* < 0.001) and motion artefacts (AC1 = 0.84, 95% CI = 0.77, 0.92, *p* < 0.001). It was slightly lower but still substantial for the patellae facing forward (AC1 = 0.76, 95% CI = 0.66, 0.86, *p* < 0.001). It was moderate for hip level (AC1 = 0.68, 95% CI = 0.57, 0.80, *p* < 0.001). Overall, the high AC1 across most assessments highlights the reliability of the EOS in ensuring consistent evaluations among different raters.

### Intra-Rater Agreement

#### Rater A

Rater A demonstrated high intra-rater agreement across the different image criteria scores (Table 3). Regarding resolution, the agreement was almost perfect for the EOS (AC1 = 0.97, 95% CI = 0.90–1.00, *p* < 0.001) and DR (AC1 = 0.93, 95% CI = 0.83–1.00, *p* < 0.001). Regarding the coverage of the area of interest, the agreement was also almost perfect for the EOS (AC1 = 0.92, 95% CI = 0.80–1.00, *p* < 0.001) and DR (AC1 = 0.83, 95% CI = 0.63–1.00, *p* < 0.001). Regarding hip level and patellae facing forward, they achieved perfect agreement (AC1 = 1.00, *p* < 0.001) for the DR and near-perfect agreement for the EOS (AC1 = 0.95, 95% CI = 0.85–1.00, *p* < 0.001). Regarding contrast and motion artefacts, their agreement was perfect for both EOS and DR images (AC1 = 1.00), highlighting consistent evaluation.

**Table 3:** Intra-rater agreement of the European criteria as applied to EOS and DR for rater A. Agreement coefficients with 95% confidence intervals (CI) and *p*-values are reported for each criterion. Rater A demonstrated excellent intra-rater agreement across both imaging modalities, with perfect agreement (coefficient = 1.00) observed for motion, contrast (EOS), and several positional criteria in DR. All ratings showed statistically significant agreement (*p* < 0.001), indicating consistent assessment by the rater across repeated evaluations

Rater A	EOS		DR	
	**Agreement coefficient (95%CI)**	***p*-value**	**Agreement coefficient (95%CI)**	***p*-value**
Resolution	0.97 (0.90, 1.00)	< 0.001	0.93 (0.83, 1.00)	< 0.001
Coverage of the area of interest	0.92 (0.80, 1.00)	< 0.001	0.83 (0.63, 1.00)	< 0.001
Hip level	0.95 (0.85, 1.00)	< 0.001	1.00	
Patellae facing forward	0.95 (0.85, 1.00)	< 0.001	1.00	
Contrast	1.00		0.92 (0.81, 1.00)	< 0.001
Motion	1.00		1.00	

### Rater B

Rater B demonstrated substantial intra-rater agreement across the various image criteria scores (Table 4). Regarding resolution, their agreement was perfect (AC1 = 1.00), indicating high consistency. Regarding the coverage of the area of interest, the agreement was higher for the EOS (AC1 = 0.85, 95% CI = 0.68–1.00, *p* < 0.001) than for the DR (AC1 = 0.69, 95% CI = 0.42–0.95, *p* < 0.001) modality. Regarding the hip level, the agreement was again higher for the EOS (AC1 = 0.85, 95% CI = 0.68–1.00, *p* < 0.001) than for the DR (AC1 = 0.64, 95% CI = 0.36–0.92, *p* < 0.001) modality. Regarding the patellae facing forward, the agreement was high for both the EOS (AC1 = 0.86, 95% CI = 0.70–1.00, *p* < 0.001) and DR (AC1 = 0.72, 95% CI = 0.49–0.97, *p* < 0.001) modalities. Regarding contrast, the agreement was almost perfect for both the EOS (AC1 = 0.94, 95% CI = 0.84–1.00, *p* < 0.001) and DR (AC1 = 0.97, 95% CI = 0.90–1.00, *p *< 0.001) modalities. Lastly, regarding motion, their agreement was almost perfect for the EOS modality (AC1 = 0.86, 95% CI = 0.70–1.00, *p* < 0.001) and perfect for the DR modality (AC1 = 1.00, *p* < 0.001).

**Table 4:** Intra-rater agreement of the European criteria as applied to EOS and DR for rater B. Agreement coefficients with 95% confidence intervals (CI) and *p*-values are presented for each criterion. Rater B demonstrated excellent intra-rater reliability for most criteria across both modalities, with perfect agreement (coefficient = 1.00) observed for resolution (EOS and DR) and motion (DR). Agreement was generally higher for EOS than for DR, particularly in positional criteria such as “coverage of the area of interest” and “hip level.” All agreement values were statistically significant (*p* < 0.001)

Rater B	EOS		DR	
	**Agreement coefficient (95%CI)**	***p*-value**	**Agreement coefficient (95%CI)**	***p*-value**
Resolution	1.00		1.00	
Coverage of the area of interest	0.85 (0.68, 1.00)	< 0.001	0.69 (0.42, 0.95)	< 0.001
Hip level	0.85 (0.68, 1.00)	< 0.001	0.64 (0.36, 0.92)	< 0.001
Patellae facing forward	0.86 (0.70, 1.00)	< 0.001	0.72 (0.49, 0.97)	< 0.001
Contrast	0.94 (0.84, 1.00)	< 0.001	0.97 (0.90, 1.00)	< 0.001
Motion	0.86 (0.70, 1.00)	< 0.001	1.00	

### Rater C

Rater C demonstrated variable intra-rater agreement across the different image criteria scores (Table 5). Regarding resolution, the agreement was perfect for the EOS modality (AC1 = 1.00) and almost perfect for the DR modality (AC1 = 0.90, 95% CI = 0.78–1.00, *p* < 0.001). Regarding the area of interest, the agreement was higher for the EOS modality (AC = 0.86, 95% CI = 0.70–1.00, *p* < 0.001) than for the DR modality (AC1 = 0.67, 95% CI = 0.41–0.94, *p* < 0.001). Regarding the hip level, the agreement was again higher for the EOS modality (AC1 = 0.77, 95% CI = 0.55–0.99, *p* < 0.001) than for the DR modality (AC1 = 0.54, 95% CI = 0.22–0.85, *p* < 0.001). Regarding the patellae facing forward, the agreement was again higher for the EOS modality (AC1 = 0.81, 95% CI = 0.61–1.00, *p* < 0.001) than for the DR modality (AC1 = 0.58, 95% CI = 0.28–0.88, *p* < 0.001). Regarding contrast, the agreement was perfect for the EOS modality (AC1 = 1.00) and almost perfect for the DR modality (AC1 = 0.97, 95% CI = 0.90–1.00, *p* < 0.001). Regarding motion artefacts, the agreement was markedly higher for the EOS modality (AC1 = 0.88, 95% CI = 0.73–1.00, *p* < 0.001) than for the DR modality (AC1 = 0.30, 95% CI = −0.03–0.68, *p* = 0.12).

**Table 5:** Intra-rater agreement of the European criteria as applied to EOS and DR for rater C. Agreement coefficients with 95% confidence intervals (CI) and *p*-values are shown for each image quality criterion. Rater C demonstrated strong intra-rater agreement for EOS across all criteria, with perfect agreement (coefficient = 1.00) for resolution and contrast. Agreement was generally lower for DR, particularly for motion (coefficient = 0.30, *p* = 0.12), which did not reach statistical significance. Other criteria in DR showed moderate to high agreement, with all remaining *p*-values < 0.001

Rater C	EOS		DR	
	**Agreement coefficient (95%CI)**	***p*-value**	**Agreement coefficient (95%CI)**	***p*-value**
Resolution	1.00		0.90 (0.78, 1.00)	< 0.001
Coverage of the area of interest	0.86 (0.70, 1.00)	< 0.001	0.67 (0.41, 0.94)	< 0.001
Hip level	0.77 (0.55, 0.99)	< 0.001	0.54 (0.22, 0.85)	< 0.001
Patellae facing forward	0.81 (0.61, 1.00)	< 0.001	0.58 (0.28, 0.88)	< 0.001
Contrast	1.00		0.97 (0.90, 1.00)	< 0.001
Motion	0.88 (0.73, 1.00)	< 0.001	0.30 (−0.03, 0.68)	0.12

### EOS diagnostic summaries

The diagnostic summaries of the EOS system and DR were compared using several key metrics: sensitivity, specificity, and AUC. These metrics provide an understanding of its reliability and diagnostic performance. True positives (TPs), true negatives (TNs), false positives (FPs), and false negatives (FNs) explain the practical benefits and limitations of each imaging modality. As there was high agreement between observers, the DR reads of the radiographer with 5 years of experience (A.A.) were used as the reference and the three observers’ EOS reads were compared to it to compute the overall TPs, TNs, FPs, and FNs, based on which the sensitivity and specificity were computed. Positive predictive value (PPV), negative predictive value (NPV), and AUC were computed.

### Resolution

**Fig. 2:**
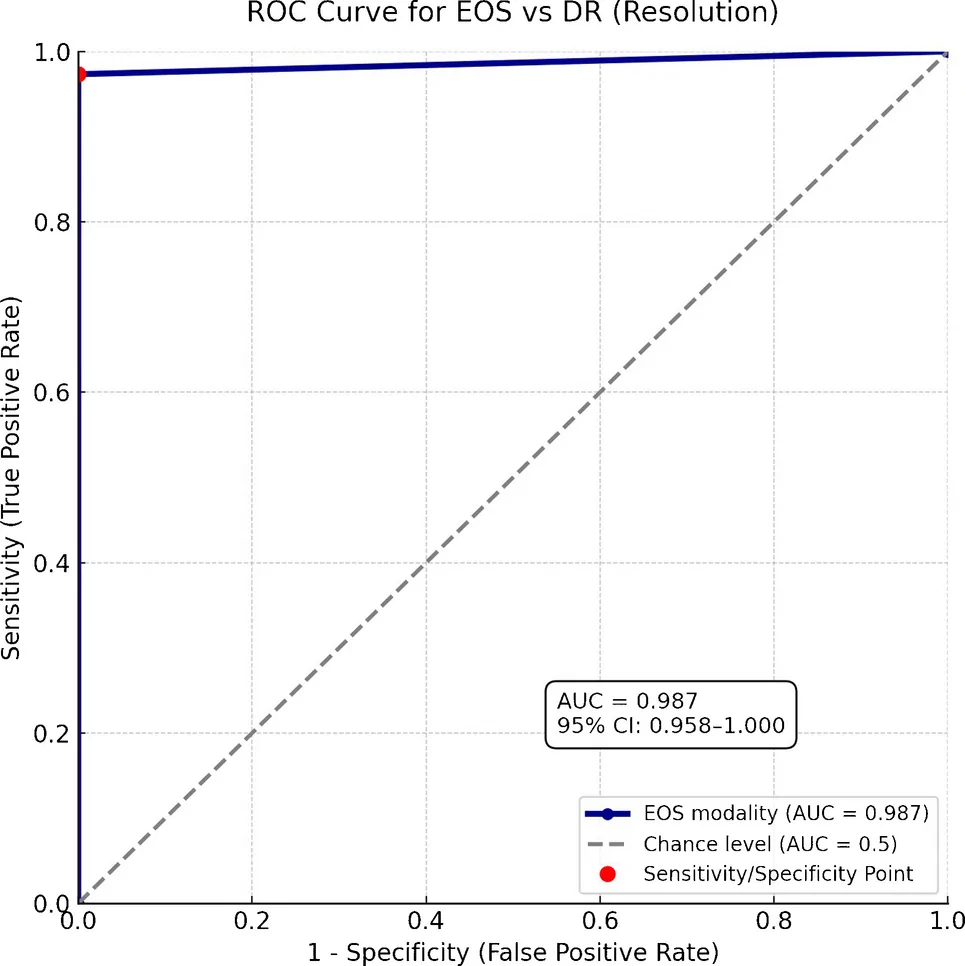
Receiver operating characteristic (ROC) curve evaluating the performance of the EOS imaging modality in distinguishing EOS from DR based on the resolution

Regarding resolution, the EOS demonstrated higher sensitivity and specificity than the DR. The EOS had 291 TPs, 8 FNs, 1 TN, and 0 FPs, resulting in a sensitivity of 97.32%, a specificity of 100.00%, and an AUC of 98.66, CI = 48.65,99.42 (Table 13). Its high sensitivity and specificity mean that the EOS is excellent at correctly identifying resolution, which is crucial for accurate diagnosis (Table 6) (Fig. 2).

**Table 6:** Diagnostic performance metrics comparing EOS to DR (resolution) across three observers, including sensitivity, specificity, AUC, PPV, and NPV with 95% confidence intervals

**EOS vs. DR **(resolution)	**TN**	**FP**	**FN**	**TP**	**Sensitivity (95% CI)**	**Specificity (95% CI)**	**AUC (95% CI)**	**PPV (95% CI)**	**NPV (95% CI)**
Observer 1	1	0	2	97	97.32% (94.80%, 98.84%)	100.00% (2.50%, 100.00%)	0.99 (0.96, 1.00)	100.00% (98.74%, 100.00%)	11.11% (5.94%, 19.85%)
Observer 2	0	0	3	97					
Observer 3	0	0	3	97					
Overall	1	0	8	291					

### Coverage of the area of interest

**Fig. 3:**
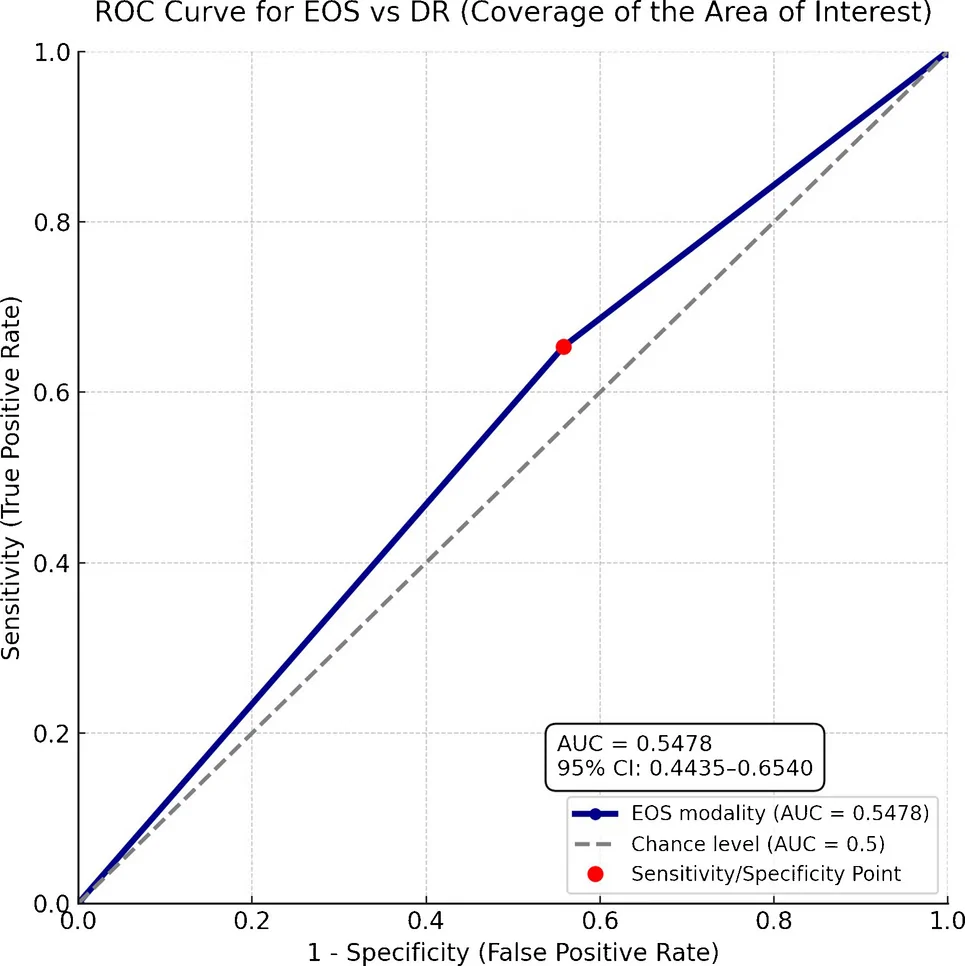
Receiver operating characteristic (ROC) curve evaluating the performance of the EOS imaging modality in distinguishing EOS from DR based on the coverage of the area of interest

In assessing the coverage of the area of interest, the EOS had 168 TPs, 89 FNs, 19 TNs, and 24 FPs, resulting in a sensitivity of 65.37, specificity of 44.19, and AUC of 0.55, CI = 0.44, 0.65. The moderate sensitivity indicates that EOS is reasonably effective in detecting coverage of the area of interest, although the lower specificity suggests that many FPs could occur (Table 7) (Fig. 3).

**Table 7:** Diagnostic performance metrics comparing EOS to DR (coverage of the area of interest) across three observers, including sensitivity, specificity, AUC, PPV, and NPV with 95% confidence intervals

**EOS vs. DR **(coverage of the area of interest)	**TN**	**FP**	**FN**	**TP**	**Sensitivity (95% CI)**	**Specificity (95% CI)**	**AUC (95% CI)**	**PPV (95% CI)**	**NPV (95% CI)**
Observer 1	9	10	27	54	65.37% (59.21%, 71.17%)	44.19% (29.08%, 60.12%)	0.55 (0.44, 0.65)	87.50% (84.10%, 90.26%)	17.59% (12.79%, 23.71%)
Observer 2	7	6	29	58					
Observer 3	3	8	33	56					
Overall	19	24	89	168					

### Hip level

**Fig. 4:**
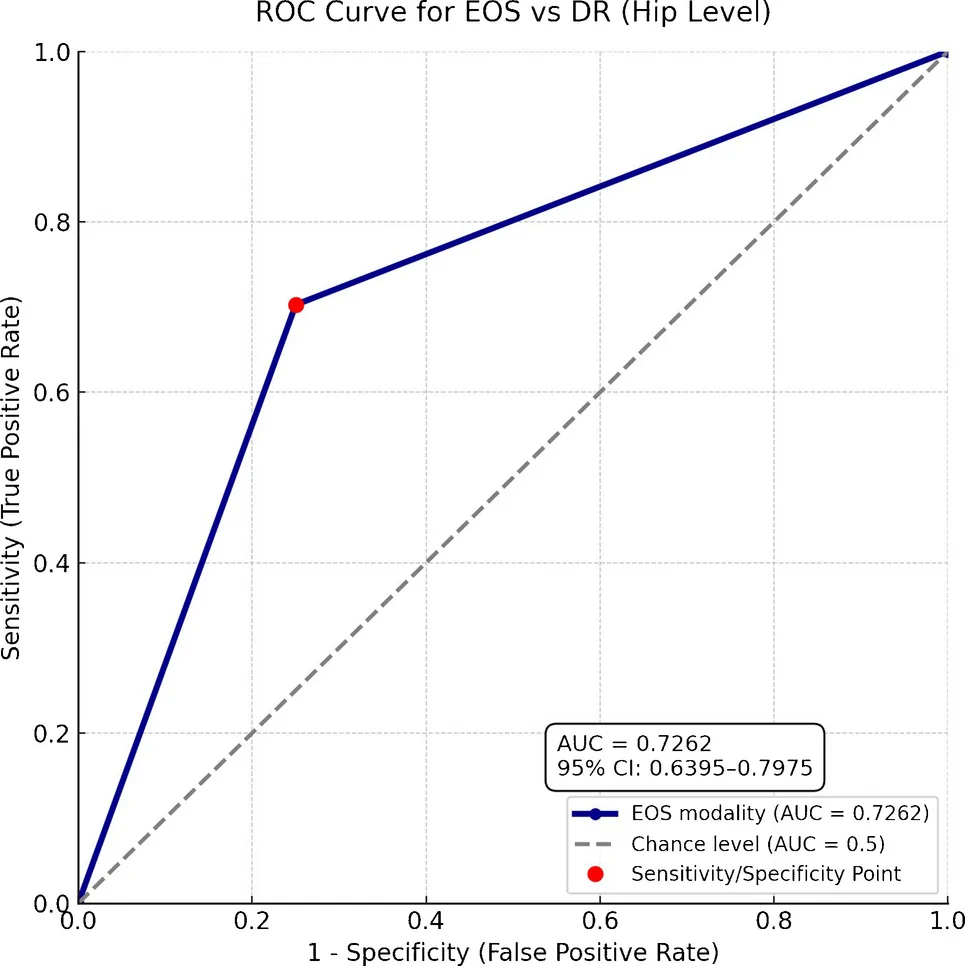
Receiver operating characteristic (ROC) curve evaluating the performance of the EOS imaging modality in distinguishing EOS from DR based on the hip level

In assessing the hip level, the EOS had 163 TPs, 69 FNs, 51 TNs, and 17 FPs, resulting in a sensitivity of 70.26%, specificity of 75.00%, and AUC of 0.73, CI = 0.64, 0.80. The moderate sensitivity and specificity underscore the EOS ability to identify the hip level correctly (Table 8) (Fig. 4).

**Table 8:** Diagnostic performance metrics comparing EOS to DR (hip level) across three observers, including sensitivity, specificity, AUC, PPV, and NPV with 95% confidence intervals

**EOS vs. DR (**hip level)	**TN**	**FP**	**FN**	**TP**	**Sensitivity (95% CI)**	**Specificity (95% CI)**	**AUC (95% CI)**	**PPV (95% CI)**	**NPV (95% CI)**
Observer 1	19	0	21	60	70.26% (63.93%, 76.06%)	75.00% (63.02%, 84.71%)	0.73 (0.64, 0.80)	90.56% (86.30%, 93.59%)	42.50% (36.75%, 48.46%)
Observer 2	18	7	22	53					
Observer 3	14	10	26	50					
Overall	51	17	69	163					

### Patellae facing forward

**Fig. 5:**
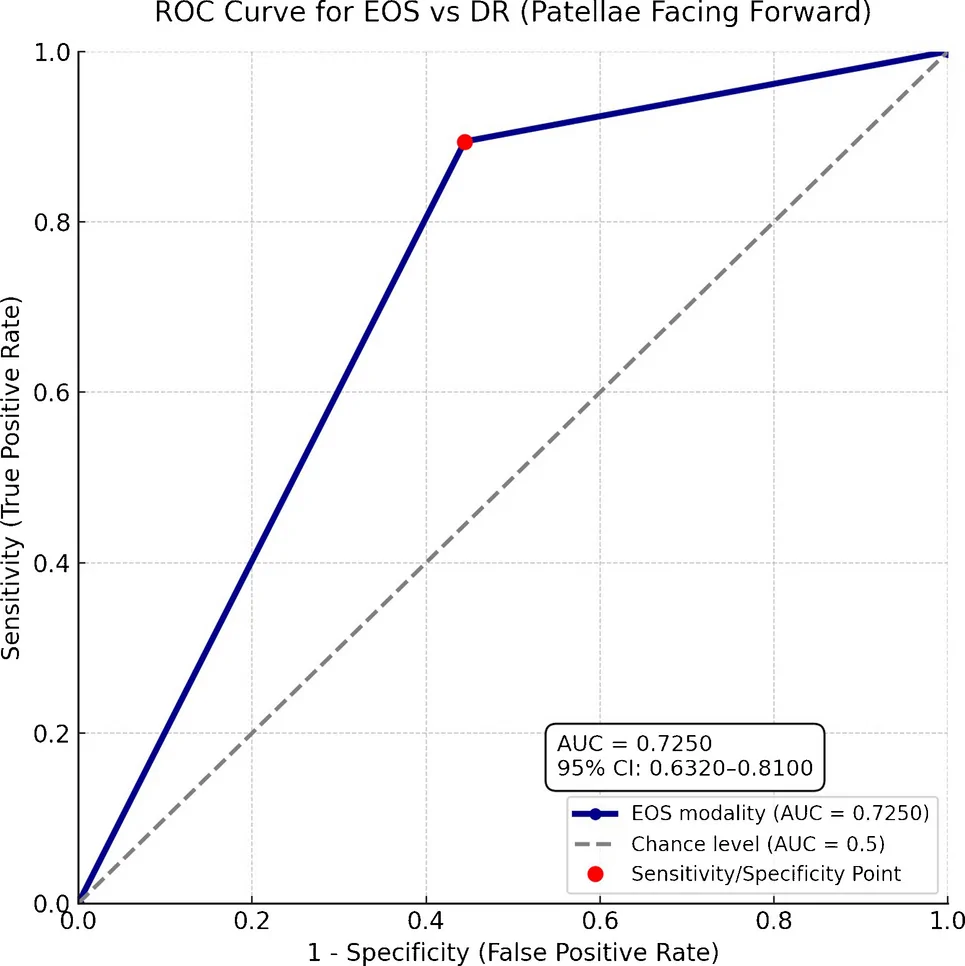
Receiver operating characteristic (ROC) curve evaluating the performance of the EOS imaging modality in distinguishing EOS from DR based on the patellae facing forward

In assessing the patellae facing forward, the EOS had 220 TPs, 26 FNs, 30 TNs, and 24 FPs, resulting in a sensitivity of 89.43%, a specificity of 55.56%, and an AUC of 0.73, CI = 0.63, 0.81. Its high sensitivity indicates that the EOS is very reliable in detecting patellae facing forward. However, its lower specificity might incorrectly rule out patellae not facing forward cases, increasing false positives and negatives, which could affect accurate patellae facing forward assessment (Table 9) (Fig. 5).

**Fig. 6:**
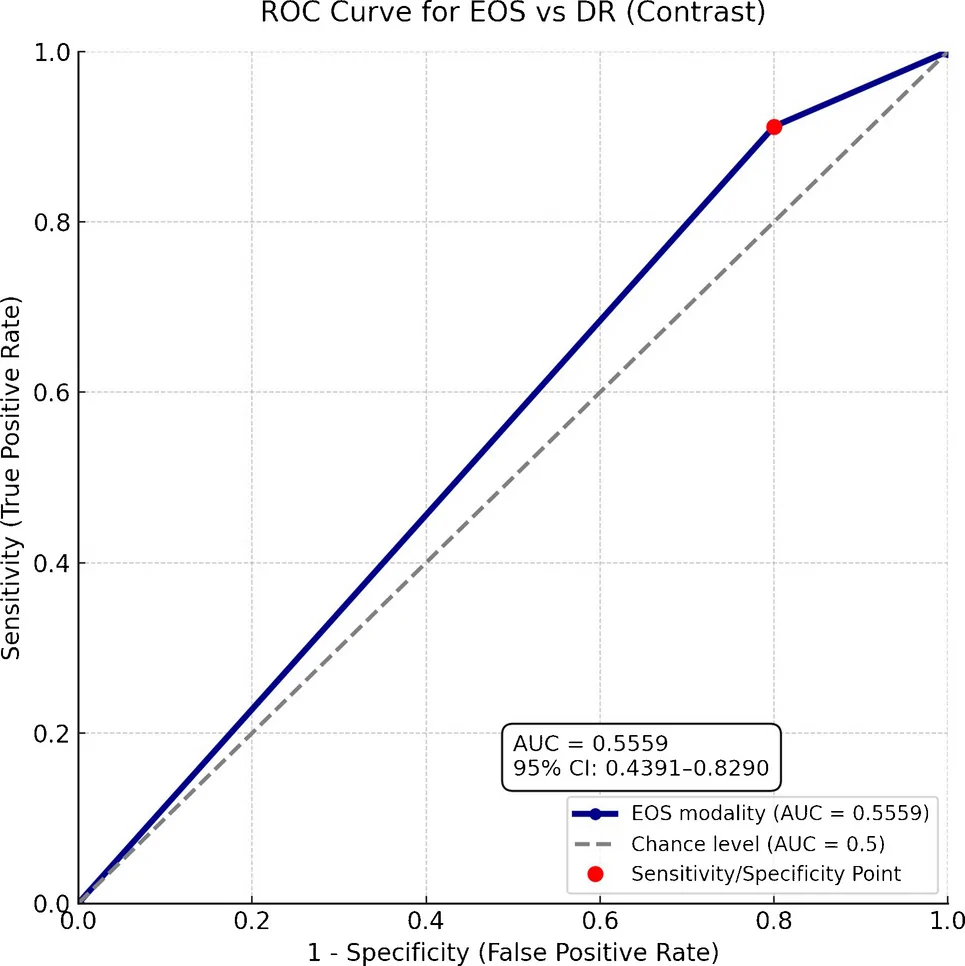
Receiver operating characteristic (ROC) curve evaluating the performance of the EOS imaging modality in distinguishing EOS from DR based on the contrast

**Table 9:** Diagnostic performance metrics comparing EOS to DR (patellae facing forward) across three observers, including sensitivity, specificity, AUC, PPV, and NPV with 95% confidence intervals

**EOS vs. DR **(patellae facing forward)	**TN**	**FP**	**FN**	**TP**	**Sensitivity (95% CI)**	**Specificity (95% CI)**	**AUC (95% CI)**	**PPV (95% CI)**	**NPV (95% CI)**
Observer 1	22	0	2	76	89.43% (84.90%, 92.98%)	55.56% (41.40%, 69.08%)	0.73 (0.63, 0.81)	90.16% (87.15%, 92.53%)	53.57% (78.62%, 87.37%)
Observer 2	2	12	12	74					
Observer 3	6	12	12	70					
Overall	30	24	26	220					

### Contrast

Regarding contrast, the EOS had 269 TPs, 26 FNs, 1 TN, and 4 FPs, resulting in a sensitivity of 91.19%, a specificity of 20.00%, and an AUC of 0.56, CI = 0.44, 0.83. Its high sensitivity ensures that almost all true cases of contrast are detected. However, its lack of specificity will likely produce FPs for this image criterion, highlighting the need to interpret results carefully (Table 10) (Fig. 6).

**Fig. 7:**
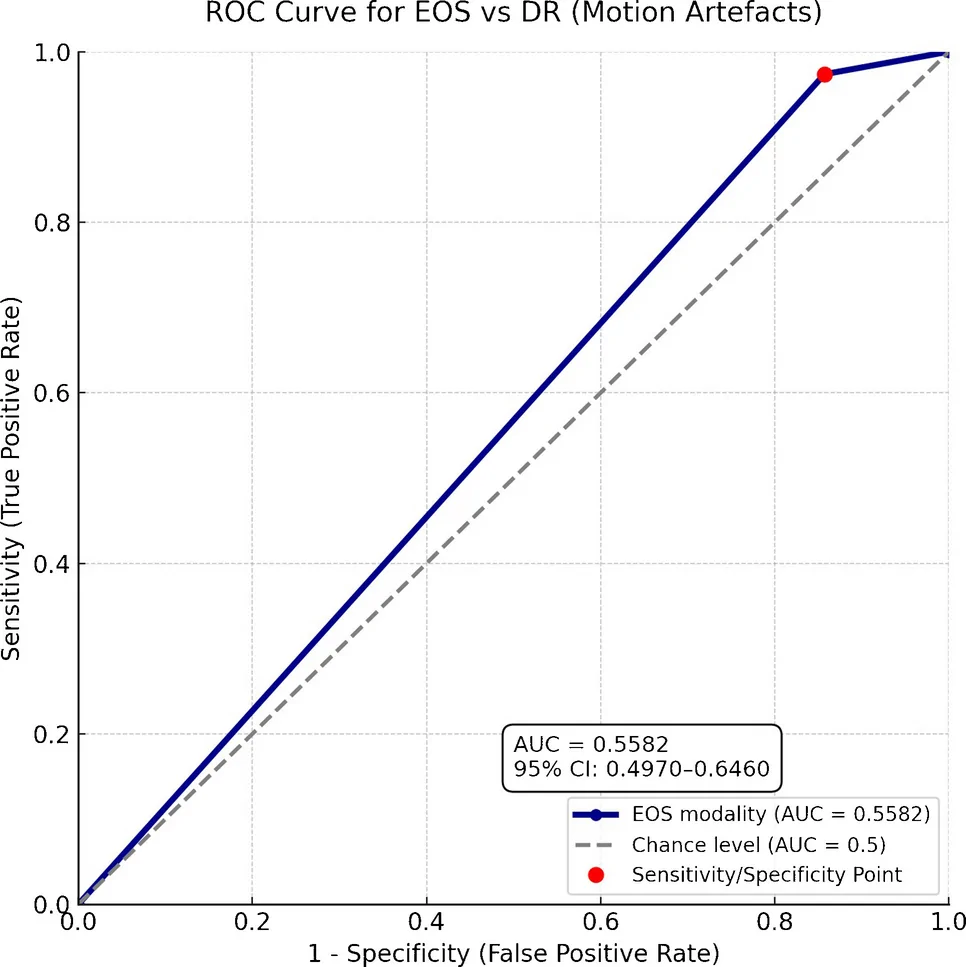
Receiver operating characteristic (ROC) curve evaluating the performance of the EOS imaging modality in distinguishing EOS from DR based on the motion artefacts

**Table 10:** Diagnostic performance metrics comparing EOS to DR (contrast) across three observers, including sensitivity, specificity, AUC, PPV, and NPV with 95% confidence intervals

**EOS vs. DR **(contrast)	**TN**	**FP**	**FN**	**TP**	**Sensitivity (95% CI)**	**Specificity (95% CI)**	**AUC (95% CI)**	**PPV (95% CI)**	**NPV (95% CI)**
Observer 1	0	1	9	90	91.19% (87.35%, 94.16%)	20.00% (0.51%, 71.64%)	0.56 (0.44, 0.83)	98.53% (97.74%, 99.05%	3.70% (0.64%, 18.74%)
Observer 2	1	3	8	88					
Observer 3	0	0	9	91					
Overall	1	4	26	269					

### Motion artefacts

Regarding motion artefacts, the EOS had 258 TP, 7 FNs, 5 TNs, and 30 FPs, resulting in a sensitivity of 97.36%, a specificity of 14.29%, and an AUC of 0.56, CI = 0.50, 0.65. Its high sensitivity is beneficial for detecting true cases of motion artefacts, although its low specificity indicates a high risk of FPs. Therefore, while the EOS effectively identifies motion artefacts, there may be cases where motion artefacts are incorrectly detected (Table 11) (Fig. 7).

**Table 11:** Diagnostic performance metrics comparing EOS to DR (motion artefacts) across three observers, including sensitivity, specificity, AUC, PPV, and NPV with 95% confidence intervals

**EOS vs. DR **(motion artefacts)	**TN**	**FP**	**FN**	**TP**	**Sensitivity (95% CI)**	**Specificity (95% CI)**	**AUC (95% CI)**	**PPV (95% CI)**	**NPV (95% CI)**
Observer 1	1	6	3	90	97.36% (94.63%, 98.93%)	14.29% (4.81%, 30.26%)	0.56 (0.50, 0.65)	89.58% (88.24%, 90.97%)	41.67% (19.33%, 68.04%)
Observer 2	2	10	2	86					
Observer 3	2	14	2	82					
Overall	5	30	7	258					

The EOS consistently demonstrated high sensitivity across various image criteria scores, ensuring that TP cases are detected effectively and reducing the likelihood of missing critical criteria, which is essential for initial diagnostic assessments. While specificity varies, the EOS generally shows better or comparable specificity to the DR in key assessments. This balance reduces FP cases and unnecessary follow-up tests, ensuring that TN cases are accurately identified. The high AUC for several image criteria indicates that the EOS provides reliable diagnostic performance, ensuring comprehensive and accurate patient evaluations, minimizing FNs and improving clinical outcomes.

By understanding the TP, TN, FP, and FN rates, clinicians can better appreciate the strengths and limitations of the EOS imaging system. This approach can aid in making informed decisions about its use in various diagnostic scenarios, ultimately improving patient care and outcomes.

### Differences between EOS and DR using McNemar’s test

We examined whether significant differences existed between the gold standard (DR) and the new modality (EOS) using McNemar’s test **(**Table 12**)**. McNemar’s test is calculated on the ratio of the discordant pairs, FP/FN. The concordant pairs TP and TN play no part in the statistical calculations, no matter how large. At the hip level, this ratio was 17/69. As a general rule, if this ratio is 3:1 or larger, it will be statistically significant. It does not make a difference if the ratio is inverted. McNemar’s test revealed significant differences between EOS and DR across most assessed parameters. Statistically significant differences were observed in resolution (chi-squared = 8.00, *p* = 0.005), coverage of the area of interest (chi-squared = 37.39, *p* < 0.001), hip-level alignment (chi-squared = 31.44, *p* < 0.001), contrast (chi-squared = 16.13, *p* < 0.001), and motion artefacts (chi-squared = 14.30, *p* < 0.001), indicating that EOS and DR perform differently in these domains. However, no significant difference was found regarding patellae facing forward (chi-squared = 0.08, *p* = 0.78), suggesting comparable performance between the two imaging modalities for this specific criterion. Overall, these findings demonstrate that EOS and DR differ significantly in most aspects of image quality, with EOS showing favourable sensitivity results in several key parameters.

**Table 12:** Comparison between the EOS imaging system and DR. Chi-squared values and corresponding p-values from McNemar’s test are presented for each European image quality criterion, comparing ratings between EOS and DR. Significant differences were observed in most criteria, including resolution, coverage of the area of interest, hip level, contrast, and motion (*p* < 0.01), indicating a preference or systematic difference between modalities. No significant difference was found for the patellae facing forward” criterion (*p* = 0.78)

**EOS vs. DR**	**McNemar’s test**	
	**Chi-squared**	***p*-value**
Resolution	8.00	0.005
Coverage of the area of interest	37.39	< 0.001
Hip level	31.44	< 0.001
Patellae facing forward	0.08	0.78
Contrast	16.13	< 0.001
Motion	14.30	< 0.001

## Discussion

Our retrospective study compared mechanical axis image quality between the EOS imaging system and DR. The agreement coefficient used in our analysis, Gwet’s AC1, is specifically designed to account for the potential dependence of ratings when the same rater evaluates the same participants at different time points or when different raters evaluate the same participants. Gwet’s AC1 incorporates a correction for chance agreement, which helps to address the issue of dependence and provides a more accurate estimate of the actual agreement among raters. Its findings show that the EOS imaging system demonstrates high reliability, including strong intra- and inter-rater agreement for most image criteria scores. Specifically, the consistency in resolution and motion evaluations emphasizes the efficacy of the EOS imaging system in providing reliable assessments.

There was also a perfect agreement between modalities and raters for the resolution and contrast image criteria. However, inter- and intra-observer agreement could have been better for some image criteria scores, such as coverage of the area of interest and positioning. Therefore, further training is needed to improve agreement.

To our knowledge, no other study has subjectively assessed the mechanical axis image quality of the EOS compared to DR. Our study found that the EOS could produce mechanical axis image quality as high as that of DR for most examined image criteria. Several studies have demonstrated that the EOS could produce images of diagnostic quality even at low doses [16, 25, 26]. One study recruited children diagnosed with scoliosis and concluded that EOS could provide high image quality for scoliosis imaging [26]. Another study using microdose EOS scans to follow-up on 25 patients with idiopathic scoliosis who were treated with braces showed that the EOS was reliable and appropriate for radiological follow-up [27]. Importantly, the images were diagnostic while adhering to the “as low as reasonably achievable” principle [28].

Our findings regarding mechanical axis image quality based on the coverage of the area of interest (pelvis to ankles) showed almost perfect inter-observer agreement between modalities and were consistent with other studies. One study reported that the EOS could be as reliable as DR in femoral mechanical axis measurements in adult patients with lower extremity deformity, with significant agreement between EOS and DR reported for all measurements [29]. In addition, a recent study concluded that the EOS could be a more accurate alternative to DR for lower extremity and implant measurements [30].

Motion artefacts are considered a disadvantage that could limit the use of the EOS imaging system [31]. These might arise because patients must remain weight-bearing during the scan, so those with lower extremity malalignment who cannot stand for long periods may become restless [19, 20]. However, this risk might be reduced because the EOS takes only 10–25 s to produce 2D biplanar radiographs [13].

Our study had several strengths, including its real-world relevance by evaluating actual patient data, which improves its applicability in clinical settings. Assessing DR and EOS images based on six image criteria scores yielded a comprehensive evaluation, and the random selection of patients minimized selection bias. The agreement among the three raters also strengthens the reliability of our findings. However, our study also had some limitations, including its retrospective design, as conducting a prospective study might provide more accurate results. In addition, the subjectivity of the observational yes or no evaluations might be less precise than quantitative measurements. Nonetheless, since our study is the first to subjectively assess the mechanical axis image quality of EOS compared to DR, it may serve as a baseline for future research.

The high AC1, sensitivity, and AUC values highlight the diagnostic image quality of the EOS for mechanical axis images, making it a valuable modality in clinical settings. As a result, the EOS can be used instead of DR in this context, offering advantages such as lower irradiation, shorter scanning times, and the capability for 3D reconstruction.

## Data Availability

The data and materials generated or analyzed during the current study are available from the corresponding author upon reasonable request.
